# Emergency department visits and disease patterns during and after the COVID-19 lockdown

**DOI:** 10.1371/journal.pone.0352275

**Published:** 2026-06-26

**Authors:** Nagham Faris, Layal Hamdar, Hani Tamim, Maha Makki, Eveline Hitti

**Affiliations:** 1 Department of Emergency Medicine, American University of Beirut Medical Center, Beirut, Lebanon; 2 Clinical Research Institute, Department of Internal Medicine, American University of Beirut Medical Center, Beirut, Lebanon; 3 College of Medicine, Alfaisal University, Riyadh, Saudi Arabia; Universitair Kinderziekenhuis Koningin Fabiola: Hopital Universitaire des Enfants Reine Fabiola, BELGIUM

## Abstract

**Background:**

The COVID-19 pandemic caused significant disruptions in healthcare systems worldwide, particularly in emergency department (ED) utilization. While much research has focused on pre-pandemic and lockdown periods, most published evidence comes from high-income settings, with fewer studies from LMICs examining the post-lockdown ED trends and diagnostic patterns.

**Aim:**

This study analyzed variations in patient volumes and shifts in disease patterns among both adult and pediatric patients presenting to the ED during and after the COVID-19 lockdown at a tertiary care center in Lebanon.

**Methods:**

A retrospective observational study was conducted using administrative data from the ED of a tertiary care medical center in Beirut, Lebanon. The study focused on adult and pediatric patients admitted between February 29, 2020, and April 14, 2022, and included a total of 69,870 patients, of whom 21.2% were pediatric cases.

**Results:**

The average age of patients decreased significantly post-lockdown (41.7 ± 24.9 vs. 37.7 ± 25.7 years, p-value <0.001), driven by a rise in pediatric visits (from 17.4% to 23.9%, p-value <0.001) demonstrating a catch-up effect. Adult volumes recovered more slowly throughout the post-lockdown period. Pediatric visits for respiratory tracts infections, COVID-19, and ear diseases surged post-lockdown (increases ranging from 303% to 355%). Adult patients showed decreases in COVID-19 and pregnancy-related visits and significant increases in injury, poisoning, digestive system diseases, and other viral infections. Post-lockdown, ED visits shifted toward lower acuity, with reduced admission rates and a slight decrease in length of stay.

**Conclusion:**

After the COVID-19 containment period, ED visits significantly increased, especially in the pediatric age group, while adult volumes recovered more slowly with distinct diagnostic shifts. These patterns in ED visits during the pandemic highlight the need for continuous ED surveillance and flexible, adaptive healthcare strategies that can rapidly scale services and adjust staffing to meet the evolving demands of both pediatric and adult populations during and after public health emergencies.

## Introduction

In December 2019, a novel coronavirus, severe acute respiratory syndrome coronavirus 2 (SARS-CoV-2), emerged and led to coronavirus disease 2019 (COVID-19), which rapidly spread globally. The COVID-19 pandemic caused significant disruptions in healthcare systems worldwide, particularly in emergency department (ED) utilization. Lockdown measures aimed at controlling viral spread led to substantial changes in healthcare-seeking behaviors, resulting in a marked decline in ED visits across various populations and regions [1,2].

Many studies have examined the effects of COVID-19 and lockdowns on ED trends, primarily by comparing pre-pandemic phases to lockdown periods. Research from multiple countries has documented significant declines in ED visits, affecting both adult and pediatric populations [3,4]. In the U.S., Hartnett et al. (2020) reported a 42% drop in ED visits during the lockdown, with the sharpest declines among children under 14. Fear of virus exposure, policy changes, and shifts in disease incidence due to social distancing and lockdown measures were major contributors to this decline [5].

In a low- and middle-income country (LMIC) like Lebanon, which has faced an ongoing financial crisis since late 2019 and where the Government Response Stringency Index reached 85, one of the highest in the world-strict lockdown measures contributed to a sharp decline in ED visits [6]. However, little research has been done on the changes in ED utilization and disease patterns that followed the lifting of lockdown measures, particularly in LMICs as opposed to high-income countries where most evidence originates. In Lebanon, our authors previously compared ED visits in Lebanon during the three months before the first identified COVID-19 case with the first three months of the pandemic, reporting a 47.2% decline in ED volumes, with the greatest drops among pediatric patients (67.8%). The steepest declines coincided with school closures and border lockdowns [7]. Compounding these challenges, the Beirut port explosion in August 2020 further strained Lebanon’s already fragile healthcare system during the lockdown period, representing an additional unmeasured stressor on ED utilization that is largely absent from studies conducted in high-income settings [8].

While several studies have compared ED utilization across pre-lockdown, lockdown, and post-lockdown phases, most have relied on aggregated surveillance data, often overlooking key demographic and socioeconomic factors that influence ED access and utilization. Additionally, many have been limited by short follow-up periods, small sample sizes, or a focus on either pediatric or adult populations with limited post-lockdown observation [9–11]. Also, most research done on post-lockdown visit trends explored presenting complaints or the primary reason for ED admission rather than final diagnoses [2,4,12,13]. Certain conditions, such as myocardial infarction, chronic ischemic heart disease, and coma, saw an increase in ED visits post-COVID-19 [14]. However, few studies have systematically documented final diagnoses using a standardized categorization framework [15]. Moreover, studies comparing lockdown and post-lockdown trends in both pediatric and adult ED visits in an LMIC setting, particularly with a focus on specific diagnoses, remain scarce. In resource-limited settings like Lebanon, where access to primary care is constrained by financial hardship and infrastructure gaps, the ED often serves as a primary point of care, making ED utilization data particularly informative for capturing post-lockdown care-seeking patterns, including potential catch-up visits among pediatric patients [7,16]

In this study, we analyzed variations in patient volumes and shifts in disease patterns among both adult and pediatric patients presenting to the ED during and after the COVID-19 lockdown at a Lebanese tertiary care center using the Clinical Classifications Software Refined (CCSR) criteria for categorization.

## Materials and methods

### Study setting and design

A retrospective observational study was conducted using administrative data of both pediatric and adult patients admitted to the American University of Beirut Medical Center’s Emergency Department (AUBMC-ED) during the lockdown and post lockdown. The lockdown period, as mandated by the Lebanese government, spanned from February 29, 2020; corresponding to the onset of official lockdown measures in Lebanon; to March 22, 2021 [17]. The lockdown included the closure of daycares, schools, universities, and all educational institutions, as well as entertainment venues. It also involved the declaration of a state of public mobilization, which included the closure of borders. The Lebanese government implemented a phased reopening strategy prior to the total lifting of all stringency measures. The post-lockdown period, as defined in our study, refers to the phase in which the Lebanese Ministry of Health mandated the total lifting of all stringency measures, which started on March 23, 2021 [17]. In our study, this phase is examined until April 14, 2022. The two study periods were of equal duration (55 weeks each), ensuring direct comparability between the lockdown and post-lockdown phases.

AUBMC is a tertiary care center in Beirut, Lebanon. The AUBMC-ED is one of the largest in the country, with 43 beds and approximately 57,000 annual visits, 28% of which are pediatric cases. There are three distinct clinical areas in the ED catering to high acuity, low acuity, and pediatric cases. Patients are triaged to specific sections based on a thorough assessment involving the Emergency Severity Index (ESI) and clinical criteria. The ESI is a five-level triage algorithm, ranging from ESI 1 (most critical) to ESI 5 (least urgent), used to prioritize patients based on acuity and anticipated resource needs. Pediatric patients exclusively receive care in the pediatric section. Those with ESI levels 1 & 2 and more complex ESI 3 cases are directed to the higher acuity area, while individuals with lower acuity ESI 3, 4 & 5 are attended to in the low acuity section. Additionally, 80% of ED patients hold insurance coverage, with the remaining being patients paying out-of-pocket.

Ethical approval was obtained from the American University of Beirut Institutional Review Board (BIO-2022–0232) on September 27, 2022. The IRB waived the requirement for informed consent due to the retrospective nature of the study and the use of a fully de-identified administrative dataset. The principal investigators and research team were granted access to the dataset on October 4, 2022, and had no access to identifiable information at any point during or after the study.

### Participant selection

All adult and pediatric patients admitted to the ED between February 29, 2020, and April 14, 2022, were included in this study. All patients included in this study had complete clinical data. Patients who left without receiving a diagnosis were excluded, as their records were insufficient for the diagnostic analysis. The final sample size was 69,870 subjects.

### Data collection

The De-identified information included the following variables: patient demographics (age and gender) and ED visit specifics such as ESI, length of stay (LOS), ED discharge status, financial guarantor, and International Classification of Diseases, 10th Revision (ICD-10) diagnosis codes. Financial guarantor was retained as a proxy for socioeconomic status, given its clinical relevance in the Lebanese healthcare context, where 80% of patients hold insurance coverage and the remainder pay out-of-pocket. The dataset was limited to the variables relevant to the study objectives; additional sociodemographic variables were not included in the analysis. The ICD-10 codes were condensed by the research team into a more concise set of categories using the CCSR for ICD-10-CM beta version, as defined by the Agency for Healthcare Research and Quality (AHRQ), without modification to the original category definitions. No alterations were made to the dataset itself; the administrative data were used as extracted from the ED information system, with the sole analytical step being the mapping of ICD-10 codes to their corresponding CCSR categories.

### Statistical analysis

All statistical analyses were performed using the Statistical Package for Social Sciences (SPSS, version 28), with a threshold of significance set at a p-value <0.05. The following variables were recoded for analysis: the study period was coded as a dichotomous variable (lockdown vs. post-lockdown) as described above; age was categorized into pediatric (1–17 years) and adult (≥18 years) groups; ED section was categorized into three areas (high acuity, low acuity, and pediatric); financial guarantor was dichotomized into self-pay and insured/third-party payment; and ED disposition was categorized into five groups (admitted, discharged, deceased, left without being seen, and transferred).

Descriptive statistics were performed using numbers and percentages for categorical variables and means with standard deviations (SD) for continuous variables. The association between lockdown and post-lockdown periods, and categorical variables was assessed using the chi-square test, while the association with continuous variables was evaluated using Student’s T-test. The examination involved calculating the percent drop in visits by diagnosis and assessing the odds ratios and 95% confidence interval (CI) for ED presentation for a specific diagnosis during both periods. Given the large number of diagnostic categories tested, the possibility of Type I error cannot be excluded, as a multiple-comparison correction was not applied; this should be considered when interpreting individual p-values.

## Results

Table 1 presents a comparison of patient demographics and ED visit characteristics during and after lockdown. During the study period, 69,870 ED visits were recorded, with 28,972 visits occurring during the lockdown phase (Feb 29, 2020 – Mar 22, 2021) and 40,898 visits post-lockdown (Mar 23, 2021 – Apr 14, 2022). The average age of patients decreased significantly post-lockdown (41.7 ± 24.9 vs. 37.7 ± 25.7 years, p-value <0.001), driven by a significant rise in pediatric visits (from 17.4% to 23.9%, p-value <0.001). Daily ED visits significantly increased post-lockdown (85.5 ± 25.3 to 108.8 ± 16.9, p-value <0.001).

**Table 1:** Comparison of patient demographics and ED visit characteristics between Lockdown and post- lockdown (N = 69,870).

Variables		Lockdown (Feb 29, 2020- Mar 22, 2021) (N = 28,972)	Post lockdown (Mar 23, 2021− 14 Apr 2022) (N = 40,898)	p-value
**Age**	Mean ± SD	41.7 ± 24.9	37.7 ± 25.7	**<0.001**
	1–17 years	5035 (17.4%)	9767 (23.9%)	**<0.001**
	18–44 years	11312 (39.0%)	15673 (38.3%)	
	45–64 years	6203 (21.4%)	7482 (18.3%)	
	65 years and older	6422 (22.2%)	7976 (19.5%)	
**Gender**	Male	15183 (52.4%)	20852 (51.0%)	**<0.001**
	Female	13789 (47.6%)	20046 (49.0%)	
**ESI**	1	163 (0.6%)	165 (0.4%)	**<0.001**
	2	3145 (10.9%)	2953 (7.2%)	
	3	24345 (84.1%)	34349 (84.1%)	
	4	935 (3.2%)	3010 (7.4%)	
	5	376 (1.3%)	486 (0.9%)	
**LOS (min)**	Mean ± SD	330. 5 ± 755.7	319.2 ± 589.5	**0.02**
**ED Disposition**	Admitted	7808 (27.0%)	8474 (20.7%)	**<0.001**
	Discharged	20804 (71.8%)	31764 (77.7%)	
	Deceased	184 (0.6%)	242 (0.6%)	
	LWBS	29 (0.1%)	242 (0.6%)	
	Transferred	147 (0.5%)	167 (0.4%)	
**Guarantor Group**	Self-Payer	6155 (21.3%)	8512 (21.0%)	0.35
	Insurance or 3rd party payment	22761 (78.7%)	32033 (79.0%)	
**Number of daily visits**	Mean ± SD	85.52 ± 25.34	108.78 ± 16.93	**<0.001**

Chi-square test used for categorical variables; Student’s T-test used for continuous variables. ESI: Emergency Severity Index; LOS: Length of Stay; LWBS: Left Without Being Seen.

Post-lockdown, the gender distribution showed a statistically significant shift, with a slight decrease in the proportion of male visits (52.4% to 51.0%) and a corresponding increase in female visits (47.6% to 49.0%), p < 0.001. The ESI distribution shifted toward less acute presentations post-lockdown, with a significant decrease in ESI 2 cases (10.9% to 7.2%, p-value<0.001) and an increase in ESI 4 cases (3.2% to 7.4%, p-value <0.001). The mean ED length of stay slightly decreased post-lockdown (330.5 ± 755.7 vs. 319.2 ± 589.5 minutes, p = 0.02). Hospital admission rates declined post-lockdown (27.0% to 20.7%, p-value <0.001), and discharge rates increased (71.8% to 77.7%, p-value<0.001). Mortality rates remained low and stable, while the proportion of patients who left without being seen (LWBS) increased post-lockdown (0.1% vs. 0.6%, p < 0.001).

Table 2 presents an age stratified comparison of patient demographics and ED visit characteristics during and post-lockdown. The post-lockdown rise in daily visits was considerably more pronounced among pediatric patients, whose daily visits nearly doubled (16.6 ± 6.7 to 28.3 ± 8.7, p < 0.001), compared to a more modest rise among adults (68.9 ± 21.3 to 80.5 ± 12.3, p < 0.001). Among adults (n = 55,068), the mean age decreased slightly post-lockdown (49.1 ± 20.8 vs. 47.6 ± 21.3, p-value<0.001). The proportion of male visits decreased while that of female visits increased (52.1% to 49.5%, 47.9% to 50.5% respectively, p-value<0.001). Among pediatric patients (N = 14,802), by contrast, the proportion of male visits increased post-lockdown (53.7% to 55.7%) while female visits declined (46.3% to 44.3%, p = 0.02); a pattern opposite to that observed in adults.

**Table 2:** Comparison of patient demographics and ED visit characteristics between Lockdown and post- lockdown stratified by age.

		Among adults N = 55,068			Among pediatrics N = 14,802		
**Variables**		**Lockdown** **(Feb 29, 2020- Mar 22, 2021)** **(N = 23,937)**	**Post lockdown** **(Mar 23, 2021− 14 Apr 2022)** **(N = 31,131)**	**p-value**	**Lockdown** **(Feb 29, 2020- Mar 22, 2021)** **(N = 5035)**	**Post lockdown** **(Mar 23, 2021− 14 Apr 2022)** **(N = 9767)**	**p-value**
**Age**	Mean ± SD	49.1 ± 20.8	47.6 ± 21.3	**<0.001**	6.5 ± 5.3	6.2 ± 5.3	**0.001**
**Gender**	Male	12479 (52.1%)	15411 (49.5%)	**<0.001**	2704 (53.7%)	5441 (55.7%)	**0.02**
	Female	11458 (47.9%)	15720 (50.5%)		2331 (46.3%)	4326 (44.3%)	
**ESI**	1	156 (0.7%)	152 (0.5%)	**<0.001**	7 (0.1%)	13 (0.1%)	**<0.001**
	2	2573 (10.8%)	2184 (7.0%)		572 (11.4%)	769 (7.9%)	
	3	20182 (84.3%)	26260 (84.4%)		4163 (82.8%)	8089 (82.9%)	
	4	750 (3.1%)	2242 (7.2%)		185 (3.7%)	768 (7.9%)	
	5	273 (1.1%)	269 (0.9%)		103 (2.0%)	117 (1.2%)	
**LOS (min)**	Mean ± SD	347.9 ± 811.2	332.2 ± 627.1	**0.01**	248.2 ± 387.2	277.7 ± 443.7	**<0.001**
**ED Disposition**	Admitted	6946 (29.0%)	7314 (23.5%)	**<0.001**	862 (17.1%)	1160 (11.9%)	**<0.001**
	Discharged	16675 (69.7%)	23302 (74.9%)		4129 (82.0%)	8462 (86.7%)	
	Deceased	180 (0.8%)	211 (0.7%)		4 (0.1%)	31 (0.3%)	
	LWBS	29 (0.1%)	198 (0.6%)		0 (0.0%)	44 (0.5%)	
	Transferred	107 (0.4%)	102 (0.3%)		40 (0.8%)	65 (0.7%)	
**Guarantor Group**	Self-Pay	5284 (22.1%)	6734 (21.8%)	0.41	871 (17.3%)	1778 (18.4%)	0.12
	Insured	18606 (77.9%)	24125 (78.2%)		4155 (82.7%)	7908 (81.6%)	
**Number of daily visits**	Mean ± SD	68.9 ± 21.3	80.5 ± 12.3	**<0.001**	16.6 ± 6.7	28.3 ± 8.7	**<0.001**

Chi-square test used for categorical variables; Student’s T-test used for continuous variables. ESI: Emergency Severity Index; LOS: Length of Stay; LWBS: Left Without Being Seen.

**Fig 1:**
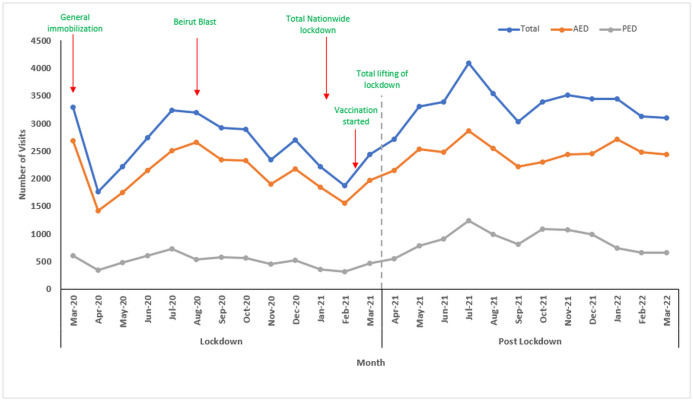
ED visits monthly trend for adults and pediatrics. Monthly ED visit volumes for adult and pediatric patients from March 2020 to March 2022. Both groups experienced a marked decline during the lockdown phase. Following the lifting of restrictions, pediatric visit volumes rose substantially before stabilizing. Adult volumes increased post-lockdown but continued to fluctuate throughout the observed recovery period. Annotated arrows indicate key events: General Mobilization, Beirut Port Explosion, Total Nationwide Lockdown, Vaccination Start, and Total Lifting of Lockdown. Total: total ED volume; AED: adult ED volume; PED: pediatric ED volume.

Acuity shifted toward lower severity in both groups. Among adults, ESI 2 cases decreased from 10.8% to 7.0%, while ESI 4 cases increased from 3.1% to 7.2% with a p < 0.001. Similarly, among pediatric patients, ESI 2 declined from 11.4% to 7.9% and ESI 4 rose from 3.7% to 7.9% with a p < 0.001). Hospital admission rates declined in both groups (adults: 29.0% to 23.5%; pediatrics: 17.1% to 11.9%, p < 0.001 for both), with corresponding rises in discharge rates (adults: 69.7% to 74.9%; pediatrics: 82.0% to 86.7%). LOS diverged between the two groups: adult LOS decreased post-lockdown (347.9 ± 811.2 vs. 332.2 ± 627.1 minutes, p = 0.01), whereas pediatric LOS increased significantly (248.2 ± 387.2 vs. 277.7 ± 443.7 minutes, p < 0.001). Monthly ED visit trends for both groups are illustrated in Fig 1.

Fig 1. illustrates monthly ED visit trends for adult and pediatric patients from March 2020 to March 2022. Both groups experienced a marked decline in visit volumes during the lockdown phase. Following the lifting of restrictions in March 2021, pediatric ED visits rose substantially, peaking in July 2021 before stabilizing. Adult ED volumes also increased post-lockdown but showed continued fluctuation without a sustained upward trajectory throughout the observation period.

Tables 3 and 4 show the percent change in visits by diagnosis as well as the odds ratios of presentation to the ED for a specific diagnosis during and post lockdown in pediatric and adult patients. Among pediatrics, the most prominent increases were seen in respiratory tract infections (303.3% increase; OR: 2.23, 95% CI: 1.95–2.56), diseases of the ear and mastoid process (343.37% increase; OR: 2.35, 95% CI: 1.85–2.99), and COVID-19 (354.84% increase; OR: 2.40, 95% CI: 1.82–3.17). Influenza also showed a notable numerical increase, though this did not reach statistical significance. A significant increase was also seen in other diseases of the respiratory system, injury and poisoning, diseases of the digestive system, diseases of the skin and subcutaneous tissue, and other viral infections. In contrast, certain infectious and parasitic diseases significantly decline (18.29 decrease; OR: 0.42 95% CI: 0.30–0.58), while mental, behavioral, and neurodevelopmental disorders did not exhibit significant changes.

**Table 3:** Percent change in ED visits by diagnosis and corresponding OR (95% CI) post Lockdown compared to lockdown among pediatrics.

CCSR	Lockdown (Feb 29, 2020- Mar 22, 2021) (N = 5035)	Post lockdown (Mar 23, 2021− 14 Apr 2022) (N = 9767)	Change (%)	OR (95% CI)	p-value
Diseases of the blood and blood forming organs and certain disorders involving the immune mechanism	184 (3.7%)	233 (2.4%)	26.63	0.65(0.53-0.79)	**<0.001**
Diseases of the circulatory system	83 (1.6%)	123 (1.3%)	48.19	0.77(0.58-1.01)	0.06
Dental diseases	217 (4.3%)	302 (3.1%)	39.17	0.71(0.60-0.85)	**<0.001**
Diseases of the digestive system	361 (7.2%)	921 (9.5%)	155.12	1.36(1.20-1.54)	**<0.001**
Diseases of the ear and mastoid process	83 (1.6%)	368 (3.8%)	343.37	2.35(1.85-2.99)	**<0.001**
Endocrine, nutritional and metabolic diseases	51 (1.0%)	65 (0.7%)	27.45	0.66(0.46-0.95)	**0.03**
External causes of morbidity	116 (2.3%)	194 (2.0%)	67.24	0.86(0.69-1.09)	0.22
Diseases of the eye and adnexa	31 (0.6%)	49 (0.5%)	58.06	0.82(0.52-1.29)	0.39
Factors influencing health status and contact with health services	228 (4.5%)	287 (3.0%)	25.88	0.64(0.54-0.77)	**<0.001**
Diseases of the genitourinary system	214 (4.3%)	240 (2.5%)	12.15	0.57(0.47-0.69)	**<0.001**
COVID 19	62 (1.2%)	282 (2.9%)	354.84	2.40(1.82-3.17)	**<0.001**
Influenza	12 (0.2%)	42 (0.4%)	250.00	1.82(0.96-3.46)	0.06
Respiratory tract infections	273 (5.4%)	1101 (11.4%)	303.30	2.23(1.95-2.56)	**<0.001**
Other viral infections	277 (5.5%)	769 (7.9%)	177.62	1.48(1.28-1.70)	**<0.001**
Other infectious and parasitic diseases	82 (1.6%)	67 (0.7%)	−18.29	0.42(0.30-0.58)	**<0.001**
Injury, poisoning and certain other consequences of external causes	916 (18.2%)	1566 (16.1%)	70.96	0.87(0.79-0.95)	**0.002**
Congenital malformations, deformations and chromosomal abnormalities	25 (0.5%)	32 (0.3%)	28.00	0.66(0.39-1.12)	0.12
Mental, behavioral and neurodevelopmental disorders	28 (0.6%)	55 (0.6%)	96.43	1.02(0.65-1.61)	0.93
Diseases of the musculoskeletal system and connective tissue	139 (2.8%)	189 (1.9%)	35.97	0.70(0.56-0.87)	**0.002**
Neoplasms	56 (1.1%)	71 (0.7%)	26.79	0.66(0.46-0.93)	**0.02**
Diseases of the nervous system	137 (2.7%)	174 (1.8%)	27.01	0.65(0.52-0.82)	**<0.001**
Certain conditions originating in the perinatal period	26 (0.5%)	37 (0.4%)	42.31	0.74(0.45-1.22)	0.23
Other diseases of the respiratory system	57 (1.1%)	126 (1.3%)	121.05	1.15(0.84-1.57)	0.39
Diseases of the skin and subcutaneous tissue	68 (1.4%)	82 (0.8%)	20.59	0.62(0.45-0.86)	**0.004**
Others	802 (15.9%	1480 (15.3%)	84.54	0.95(0.86-1.04)	0.29
Fever	507 (10.1%)	845 (8.7%)	66.67	0.85(0.76-0.96)	**0.007**

OR: Odds Ratio; CI: Confidence Interval; CCSR: Clinical Classifications Software Refined. For all comparisons, the reference group is patients who did not present with the diagnosis under consideration.

**Table 4:** Percent change in ED visits by diagnosis and corresponding OR (95% CI) post Lockdown compared to lockdown among adults.

CCSR	Lockdown (Feb 29, 2020- Mar 22, 2021) (N = 23,937)	Post lockdown (Mar 23, 2021− 14 Apr 2022) (N = 31,131)	Change (%)	OR (95% CI)	p-value
Diseases of the blood and blood forming organs and certain disorders involving the immune mechanism	274 (1.1%)	363 (1.2%)	32.48	1.03 (0.88–1.20)	0.74
Diseases of the circulatory system	2397 (10.0%)	2751 (8.9%)	14.77	0.88 (0.83–0.93)	**<0.001**
Dental diseases	161 (0.7%)	243 (0.8%)	50.93	1.17 (0.96–1.43)	0.12
Diseases of the digestive system	1683 (7.0%)	2702 (8.8%)	60.55	1.27 (1.19–1.35)	**<0.001**
Diseases of the ear and mastoid process	270 (1.1%)	327 (1.1%)	21.11	0.94 (0.80–1.10)	0.44
Endocrine, nutritional and metabolic diseases	248 (1.0%)	331 (1.1%)	33.47	1.04 (0.88–1.22)	0.68
External causes of morbidity	360 (1.5%)	574 (1.9%)	59.44	1.24 (1.09–1.42)	**0.001**
Diseases of the eye and adnexa	210 (0.9%)	268 (0.9%)	27.62	0.99 (0.83–1.19)	0.91
Factors influencing health status and contact with health services	643 (2.7%)	607 (2.0%)	−5.60	0.73 (0.65–0.81)	**<0.001**
Diseases of the genitourinary system	1781 (7.4%)	2155 (7.0%)	21.00	0.93 (0.87–1.00)	**0.04**
COVID 19	1678 (7.0%)	1504 (4.9%)	−10.37	0.68 (0.63–0.73)	**<0.001**
Influenza	1196 (5.0%)	1758 (5.7%)	46.99	1.15 (1.06–1.24)	**<0.001**
Respiratory tract infections	22 (0.1%)	36 (0.1%)	63.64	1.27 (0.75–2.16)	0.38
Other viral infections	490 (2.0%)	791 (2.6%)	61.43	1.26 (1.12–1.41)	**<0.001**
Other infectious and parasitic diseases	446 (1.9%)	502 (1.6%)	12.56	0.87 (0.77–0.99)	**0.03**
Injury, poisoning and certain other consequences of external causes	3022 (12.6%)	4522 (14.6%)	49.64	1.19 (1.13–1.25)	**<0.001**
Congenital malformations, deformations and chromosomal abnormalities	8 (0.0%)	11 (0.0%)	37.50	1.07 (0.43–2.65)	0.89
Mental, behavioral and neurodevelopmental disorders	476 (2.0%)	652 (2.1%)	36.97	1.06 (0.94–1.20)	0.31
Diseases of the musculoskeletal system and connective tissue	1456 (6.1%)	1895 (6.1%)	30.15	1.01 (0.94–1.08)	0.79
Neoplasms	267 (1.1%)	386 (1.3%)	44.57	1.12 (0.96–1.31)	0.15
Diseases of the nervous system	774 (3.2%)	944 (3.1%)	21.96	0.94 (0.86–1.04)	0.24
Pregnancy, childbirth and the puerperium	182 (0.8%)	137 (0.4%)	−24.73	0.58 (0.47–0.73)	**<0.001**
Other diseases of the respiratory system	288 (1.2%)	401 (1.3%)	39.24	1.08 (0.93–1.26)	0.32
Diseases of the skin and subcutaneous tissue	377 (1.6%)	490 (1.6%)	29.97	1.01 (0.88–1.15)	0.91
Others	4663 (19.5%)	6002 (19.4%)	28.72	1.00 (0.96–1.04)	0.90
Fever	564 (2.4%)	521 (1.7%)	−7.62	0.71 (0.63–0.80)	**<0.001**

OR: Odds Ratio; CI: Confidence Interval; CCSR: Clinical Classifications Software Refined. For all comparisons, the reference group is patients who did not present with the diagnosis under consideration.

Among adults, diagnostic shifts were less pronounced. While respiratory tract infections did not show a statistically significant change among adults (63.64% increase; OR: 1.27, 95% CI: 0.75–2.16, p = 0.38), significant increases were observed in related respiratory categories, including influenza (47.0% increase; OR: 1.15, 95% CI: 1.06–1.24, p < 0.001) and other viral infections (61.4% increase; OR: 1.26, 95% CI: 1.12–1.41, p < 0.001). External causes of morbidity, diseases of the digestive system, and injury and poisoning-related visits, also showed significant increases. The most notable drops were in pregnancy, childbirth, and puerperium (24.7% decrease; OR: 0.58, 95% CI: 0.47–0.73, p < 0.001), COVID-19-related visits (10.4% decrease; OR: 0.68, 95% CI: 0.63–0.73, p < 0.001), and fever (7.6% decrease; OR: 0.71, 95% CI: 0.63–0.80, p < 0.001). Non-significant changes were observed in categories such as dental diseases, eye and adnexa diseases, musculoskeletal diseases, nervous system diseases, neoplasms, and skin diseases.

## Discussion

This study observed ED trends during and after the COVID-19 lockdown at the largest tertiary care center in Beirut, Lebanon, revealing notable shifts in patient demographics, acuity levels, and disease presentations. A significant increase in overall ED volumes was observed post-lockdown, with a marked rise in pediatric visits. While adult ED visits also increased, the change was more moderate. High-acuity presentations declined across all age groups, while low-acuity cases rose. Diagnostically, pediatric patients showed substantial increases in respiratory tract infections, COVID-19, and ear and mastoid process diseases. Adult patients, on the other hand, showed significant increases in injury and poisoning, diseases of the digestive system, external causes of morbidity, and other viral infections and influenza, and notable decreases in COVID-19-related visits, pregnancy-related visits, and fever.

This study offers several methodological distinctions from existing literature. First, while prior research often focused exclusively on either the lockdown or pre-pandemic phases and evaluated shorter timeframes, typically lasting three to six months [18,19], our study spans both the lockdown and post-lockdown periods, covering a longer duration of nearly two years. Second, whereas most existing studies limited their analysis to either adult or pediatric populations [4,10,13,20], our study simultaneously assessed both groups allowing cross population comparison. Third, many previous studies grouped diagnoses into broad or custom categories without relying on standardized classification systems, limiting cross-study comparability [4,12,15]. In our study, we used CCSR to categorize diagnoses, which enhances the robustness of our findings. Finally, our inclusion of patient-level variables, such as age, sex, and financial guarantor as a proxy for socioeconomic status, adds further depth, contrasting with studies that rely primarily on surveillance data without linking clinical profiles to utilization trends [4,11,21].

Following the lifting of lockdown measures in Lebanon, pediatric ED visits rebounded sharply. This “catch-up” effect likely reflects both renewed transmission of communicable diseases following the reopening of schools and childcare centers, and previously deferred care during the stricter lockdown phase [9,18]. Similar rebound patterns have been reported internationally, including in Italy [9,18], the Netherlands [16], and the United States [10,21], where pediatric ED volumes recovered following the easing of restrictions. In the Netherlands specifically, ED volumes, including pediatric cases, rose as the Oxford Stringency Index (OSI) declined [16]. However, in contrast to the partial recovery seen in Dutch hospitals, where pediatric visits remained suppressed following the easing of restrictions [16], our data show a more rapid and robust post-lockdown rebound in pediatric ED volumes, which rose substantially and stabilized at higher levels by mid-to-late 2021. This more pronounced rebound may be due to limited full adherence to lockdown measures, and to the ED serving as the primary point of care for pediatric patients in Lebanon, making the post-lockdown surge fully captured in ED utilization data [7]. The divergence in post-lockdown recovery trajectories between pediatric and adult patients warrants further examination. The reopening of schools and childcare facilities likely played a key role by facilitating the spread of communicable diseases. In contrast to adults, whose COVID-19-related visits declined, possibly due to vaccination efforts, reduced ED testing, and widespread availability of at-home testing kits, pediatric COVID-19 visits rose, likely due to greater exposure in school environments and lower vaccination rates in children at the time. This difference in post-lockdown utilization patterns between children and adults is further reflected in the temporal trends observed in our data (Fig 1). Pediatric ED visits increased steadily following the easing of restrictions, peaking in July 2021, likely reflecting a compounded effect of seasonal summer surges and the post-lockdown rebound in communicable disease exposure, before stabilizing through the remainder of the observation period. In contrast, adult ED volumes showed a more gradual and fluctuating recovery throughout the post-lockdown period. Several factors may explain this slower adult recovery. Lebanon’s ongoing financial crisis, which predated the pandemic and intensified during it, likely deterred many adults from seeking non-urgent ED care due to increased out-of-pocket costs and reduced insurance coverage [22]. Persistent fear of infection, particularly among older adults with comorbidities, may have further suppressed ED utilization. Additionally, the expanded availability of telemedicine and outpatient alternatives during the post-lockdown period may have redirected some adult patients away from the ED for lower-acuity conditions [23]. Together, these factors may explain why adult LOS decreased post-lockdown, reflecting a shift toward less complex presentations, while pediatric LOS increased, likely due to higher volumes and crowding in the pediatric ED.

During the lockdown, fear of the virus caused many people to avoid seeking care, leading to delayed presentations at more advanced stages and higher severities [22]. However, after the lockdown was lifted and fear of the virus diminished, there was a shift in ED acuity levels. We observed a decrease in ESI 2 cases and an increase in ESI 4 visits, suggesting a rise in lower-acuity cases and conditions with less urgent needs. This change was also accompanied by an increase in cases related to bacterial infections and external causes like injuries. Additionally, there was a rise in discharge rates post-lockdown, likely reflecting the increase in lower-acuity visits, as people became more willing to seek care for conditions that typically require less intensive treatment and shorter ED stays.

Diagnostic patterns after lockdown varied noticeably between children and adults. Among children, there was a sharp rise in infectious diseases like respiratory tract infections and ear infections, likely driven by increased exposure to viruses in school and daycare settings. The concurrent surge across multiple pediatric infectious disease categories is further consistent with the concept of ‘immunity debt,’ whereby reduced exposure to circulating pathogens during the lockdown period led to waning immunity in the pediatric population, resulting in a broad post-lockdown surge in communicable diseases upon school reopening [24,25]. In contrast, adults showed a distinct pattern of diagnostic shifts, with fewer categories reaching significance compared to pediatrics. Significant increases were observed in injury and poisoning, digestive system diseases, external causes of morbidity, and other viral infections and influenza, while COVID-19-related visits, pregnancy-related visits, fever, and circulatory system diseases declined. The increases in injury, poisoning, and external causes likely reflect adults returning to work, commuting, and other activities that carry physical risk. The observed increases in other viral infections and influenza may additionally reflect post-COVID immune dysregulation, whereby prior COVID-19 infection may have increased susceptibility to other circulating pathogens [26], though our administrative dataset does not allow direct assessment of this relationship.

The significant fluctuations in ED visits and changes in patient demographics and disease spectrum underscore the difficulty of managing healthcare services during a pandemic. These fluctuations underscore the need for dynamic ED staffing and surge-capacity models that can be scaled up or down based on observed demand. In parallel, real-time ED surveillance dashboards (e.g., tracking arrivals, triage acuity, and key diagnostic syndromes) can help detect early shifts in volume and case-mix and support faster operational decisions during public health emergencies. To better prepare for future challenges, healthcare systems need flexible, adaptive strategies that can quickly respond to changing circumstances. With an increase in lower acuity visits post-lockdown, it is essential to strengthen healthcare capacity to manage these cases, perhaps through expanding outpatient care or telemedicine options. Investing in healthcare infrastructure and workforce training will ensure systems can handle surges during crises. Finally, preparedness plans should be in place to quickly scale services during public health emergencies, ensuring the system is always ready to meet evolving healthcare needs.

### Limitations

This study has several limitations that should be considered when interpreting the findings. First, it is based on data from a single tertiary care center in Beirut, which may limit the generalizability of the results to other settings within Lebanon or the broader region. In addition, we did not have access to external data to quantify changes in the hospital’s catchment area or referral patterns over the study period. Any pandemic-related shifts in care-seeking routes or access to nearby facilities could have altered the case-mix seen at this tertiary center and may affect the magnitude of observed trends. As this study relied on administrative data, the research team had no control over which variables were collected or how they were recorded by the ED information system. Also, the retrospective design of the study introduces inherent limitations related to data completeness and potential unmeasured confounders, including the Beirut port explosion of August 2020, Lebanon’s ongoing financial crisis, changes in insurance coverage patterns, and pandemic-related shifts in care-seeking behavior that cannot be fully accounted for in administrative data. Given the large number of diagnostic categories tested, the possibility of Type I error cannot be excluded, as a multiple-comparison correction was not applied. Lastly, pre-pandemic ED volumes were not re-analyzed in this manuscript because the dataset begins on Feb 29, 2020; pre-pandemic comparisons for the same institution have been reported previously [7]. Additionally, the dichotomous comparison of lockdown versus post-lockdown periods, while methodologically justified, may obscure seasonal trends within each phase. Furthermore, unmeasured policy-level factors, including national health directives and changes in institutional protocols during the pandemic, may have influenced ED utilization patterns in ways that cannot be fully captured in administrative data.

## Conclusion

In conclusion, this study documents shifts in ED utilization patterns, patient demographics, and disease presentations during and following the COVID-19 lockdown period at a major tertiary care center in Lebanon. The marked post-lockdown rebound in pediatric visits, the divergent recovery trajectories between adult and pediatric populations, and the diagnosis-specific changes observed underscore the complexity of post-pandemic healthcare demand. These findings highlight the importance of continuous ED surveillance and flexible, adaptive resource allocation strategies to effectively manage healthcare systems during and after public health emergencies in resource-limited settings.
